# Microfiltration Process by Inorganic Membranes for Clarification of TongBi Liquor

**DOI:** 10.3390/molecules17021319

**Published:** 2012-02-01

**Authors:** Bo Li, Minyan Huang, Tingming Fu, Linmei Pan, Weiwei Yao, Liwei Guo

**Affiliations:** 1 Key Laboratory of Chinese Traditional Medicine Compound Separation Engineering, Nanjing University of Chinese Medicine, Nanjing 210029, China; Email: zodlee@gmail.com (B.L.); futingming@gmail.com (T.F.); linmeip@yahoo.com.cn (L.P.); yww0715@yahoo.com.cn (W.Y.); 2 The First People’s Hospital of Nantong, Nantong 226001, China; Email: 352500439@qq.com

**Keywords:** Tongbi liquor, microfiltration, ceramic membrane, liquor quality, ultrasonic field, Chinese Herbal Medicine

## Abstract

Membrane separation is an alternative separation technology to the conventional method of filtration. Hence, it has attracted use in the purification and concentration of Chinese Herbal Medicine Extracts (CHMEs). The purpose of this work was to study the process of microfiltration of Tongbi liquor (TBL), a popular Chinese herbal drink, using ceramic membranes. Zirconium oxide and aluminum oxide membranes with pore mean sizes of 0.2 μm and 0.05 μm, respectively, are used for comparisons in terms of flux, transmittance of the ingredients, physical-chemical parameters, removal of macromolecular materials and fouling resistance. The results show that 0.2 μm zirconium oxide membrane is more suitable. The stable permeate flux reaches 135 L·h^−1^·m^−2^, the cumulative transmittance of the indicator is 65.53%. Macromolecular materials, such as starch, protein, tannin, pectin and total solids were largely eliminated in retentate after filtration using 0.2 μm ZrO_2_ ceramic membrane, resulting in clearer TBL. Moreover, this work also reveals that continuous ultrasound could strengthen membrane process that the permeate flux increases significantly. This work demonstrates that the purification of CHME with ceramic membranes is possible and yielded excellent results.

## 1. Introduction

Tongbi liquor (TBL), a liquid extract of five common Chinese herbals (*Radix aconiti kusnezoffii praeparata*, *Angelica sinensis*, *Syzygium aromaticum*, *Rhizoma alpiniae officinarum* and *Cortex Acan thopanacis Gracilistyli*), is a very popular herbal drink in China, commonly used to treat diseases such as rheumatism, rheumatoid arthritis, sciatica, and bone hyperplasia. However, a growing number of consumers are expressing concerns about the control of quality during the process of production and storage. 

During the preparation of TBL, both active ingredients and non-bioactive substances are extracted simultaneously. The non-bioactive substances are usually macromolecular materials such as starch, pectin, and organic polymer that precipitate easily during production and storage, resulting in poor pharmaceutical performances. Therefore, it is vital to remove these non-bioactive substances from raw extracts by using an appropriate purification/separation method.

The conventional medicinal liquor/wine refining is carried out by using bentonite clay [1,2] diatomeaceous earth [3], flocculant [4], refrigeration [5] and so on. Since the active ingredients are low molecular materials with molecular weights of less than 1000 Dalton [6] that can be absorbed easily, these refining methods usually often cause significant and unexpected yield losses.

Membrane separation technique is a physical separation technology which includes microfiltration (MF), ultrafiltration (UF), nanofiltration (NF) and reverse osmosis (RO) techniques. It is a powerful tool for separating different types of molecules with different molecular weights. In general, membrane separations can operate under mild conditions of temperature, pressure and shear stress, therefore preserving the biological activity of the compounds to be recovered and the properties of the original product; they do not require any extraction mass agents such as solvents, avoiding product contaminations and the need for subsequent purification [7]. The purpose of this work was a theoretical and experimental study on the microfiltration processes to produce a stable TBL of high nutritional properties and color quality by using ceramic membranes. However, filtration has not been considered as a simple aesthetic operation. Rather, it should be used to made raw liquid perfectly clear by elimination of all the suspended solids and of the excess of those components such as starch, protein, pectin and tannins which represent an instability factor during storage. However, there are almost no scientific references dealing with the treatment of TBL by membrane processes. Considering the health promoting compounds of the liquor, efforts should be devoted to evaluate safe processes for their recovery avoiding toxic solvents. 

Although membrane technology has experienced important development in the last years, being more and more employed as an alternative to conventional separation processes, its high cost of application, concentration polarization and membrane fouling are still significant factors restraining its development. Therefore, it is necessary to explore appropriate methods to regenerate membranes or control membrane fouling. Randon *et al*. [8] linked phosphoric acid and alkylphosphonic acid to the surface of titania and zirconia membranes to modify their surface properties. The flux of modified membranes could be increased by 40% or more and Bovine Serum Albumin (BSA) rejection was also increased. Lehman *et al*. [9] analyzed the effect of ozone and coagulation pretreatment on reducing the membrane fouling. This study demonstrated that ozone treatment is effective at degrading colloidal natural organic matter of wastewater which is likely responsible for the majority of membrane fouling. Wakeman *et al*. [10] studied the effects of electric and acoustic fields on constant pressure filtration. Experimental results demonstrated that electric field is more effective at improving filtration characteristics. 

In the present work, the TBL was microfiltered using zirconium oxide and aluminum oxide ceramic membranes of pore diameter of 0.2 μm and 0.05 μm. The objectives of this study were to understand the effects of pore diameter on permeate flux, active ingredients, physico-chemical parameters, and macromolecular materials. Moreover, this work investigated the effect of ultrasonic field on microfiltration process by 0.2 μm ZrO_2_ membrane.

## 2. Results and Discussion

### 2.1. Effect of Membrane Type

**Figure 1:**
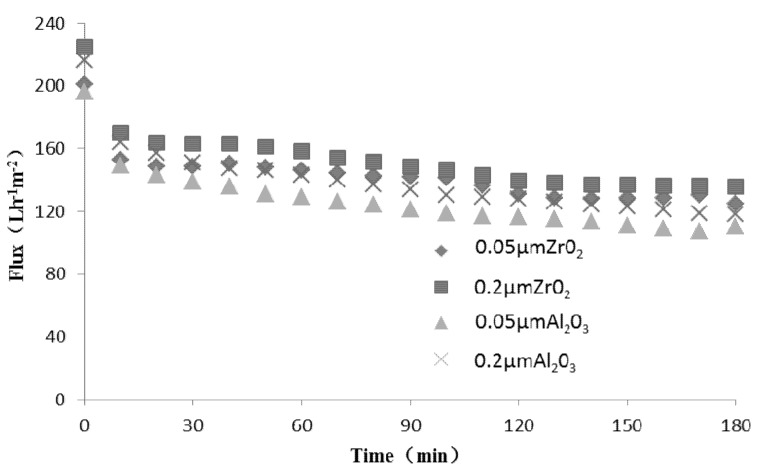
Influence of membrane on Tongbi liquor permeate flux.

Figure 1 shows the plots of fluxes along with operation time for TBL with 0.2 μm, 0.05 μm Al_2_O_3_ and ZrO_2_ membranes. It is curious that for the four types of membrane, the stable permeate flux is comparatively higher than for other biological fluids such as beer and secondary municipal waste water [11,12,13,14], the main reason may be the use of alcoholic spirit as the solvent to reconstitute the Tongbi liquor, rather than use of water, the smaller viscosity may leads to smaller membrane fouling.

In terms of dynamic changes during the whole MF process, for the four types of membrane, permeate flux declined sharply in the first 10 min and then slowly dropped. According to Howell and Velicangil [15], during the first 10 min of MF, solute is adsorbed on the membrane surface causing a 30% drop in flux and subsequently, decline in flux due to a reversible fouling caused by formation of gel layer. The MF of TBL with four types of membrane meets the regular pattern described above. On the other hand, the decrease of flux for 0.2 μm ZrO_2_ is moderate throughout the MF process.

In order to observe the dynamic changes in the process of MF, permeate was collected after the cumulative permeate volume accounted for 1/9, 2/9, 3/9 (mid-microfiltration), 4/9, 5/9, and 6/9 (end of microfiltration) of the raw liquor volume. The ferulic acid content in each permeate fraction was analyzed and the results are shown in Table 1.

**Table 1:** Dynamic transmittance of ferulic acid in TBL.

Sample	0.2 μm ZrO_2_	0.05 μm ZrO_2_	0.2 μm Al_2_O_3_	0.05 μm Al_2_O_3_
Permeate 1	10.09	10.06	9.53	9.53
Permeate 2	9.88	10.21	10.01	9.88
Permeate 3	10.51	9.53	9.88	10.22
Permeate 4	12.57	11.88	11.22	10.66
Permeate 5	11.21	10.57	10.88	10.23
Permeate 6	11.27	10.55	10.64	10.51
Cumulative Transmittance	65.53	62.80	62.16	61.03

The total transmittances of ferulic acid for the four membranes were in the range of 61.03% ~ 65.53%, respectively. Since the total volume of permeate is 6/9 of the raw liquor volume, the theoretical cumulative transmittance of ferulic acid content in the permeate should be 66.67%. The experimental results showed that all membranes had some losses, however, the cumulative transmittance of ferulic acid from the 0.2 μm ZrO_2_ membrane was higher than that of the other one.

In terms of dynamic changes of every instantaneous transmittance, it is curious that the results follow some laws and patterns. For the four membranes, the value was lower at the beginning of filtration, and the maximum values of instantaneous transmittance for membranes were observed in permeate 4 (mid-microfiltration). There was a sudden increase in instantaneous transmittance from all membranes in permeate 4; the reason of this is under study. The hypothesis is at the beginning of the filtration, a gel layer is formed rapidly so that some ferulic acid and other active ingredient are rejected or absorbed by the membrane and the gel layer. However, for the membrane and the gel layer, the maximum of adsorption is limited. As the process of filtration, desorption and adsorption become a dynamic equilibrium, the result shows that the balance may lie in the middle of permeate 3 and permeate 4 results suggested that MF process should be optimized according to the fluctuations in transmittance of active ingredients. For example, the lowest point of transmittance can be chosen as the liquid feed point in order to lower the concentration of the retentate, thereby reducing the gel layer and restoring high membrane separation efficiency.

After repeated experiments, the results are basically the same. Table 2 shows the applicability of the four membranes for separation of TBL. Focus on the permeation flux and transmittance, comparing the membrane material, the result of flux and the transmittance of ferulic acid shows that the ZrO_2_ membrane is better than the Al_2_O_3_ membrane, compared the declination of flux (FD) with time, the FD of ZrO_2_ is smaller than Al_2_O_3_. For the same pore size, the flux of ZrO_2_ is 9% ~ 12% higher than Al_2_O_3_; and the transmittance of ZrO_2_ is 3% ~ 5% higher than Al_2_O_3_ (in all the repeated experiments), which indicates that the ZrO_2_ membrane is suitable for TBL. Analysis of the permeate characteristics shows that large flux was accompanied by a higher transmittance of total active ingredients and a lower fouling. From the values of Table 2, it may be clearly seen that 0.2 μm ZrO_2_ membrane performs the best which is followed by 0.05 μm ZrO_2_membrane. Thus, 0.2 μm ZrO_2_ membrane may be recommended out of the four membranes for industrial application.

**Table 2:** Comparison of the performance of different membranes.

Membranes	Permeate Flux (L·h^−1^·m^−2^)	Cumulative Transmittance (%)
0.2 μm ZrO_2_	135	65.53
0.05 μm ZrO_2_	118	62.80
0.2 μm Al_2_O_3_	124	62.16
0.05 μm Al_2_O_3_	107	61.03

### 2.2. Macromolecular Materials and Total Solids Analysis

Since macromolecular materials and total solids are the cause of the precipitate, haze and membrane fouling, removal of these materials is crucial to improve the TBL quality and prolong the shelf-time of liquor [16,17]. As we can see from Table 3, generally, after the filtration, the macromolecular materials and total solids have been removed to some extent, in addition, the removal of the starch and pectin is more satisfactory than the others (more than 50%). The transport rate of macromolecules depends primarily on the particle/pore size ratio and complex particle–pore interactions [17]. As far as we are concerned, the reason why different macromolecular materials have different rejections is that the different compounds have different structures. For example, starch and pectin are usually often catenary shaped so that they will be easily rejected while proteins are usually ball-shaped. 

**Table 3:** Removal of macromolecular materials and total solids.

	0.2 μm ZrO_2_	0.05 μm ZrO_2_	0.2 μm Al_2_O_3_	0.05 μm Al_2_O_3_
Starch (%)	73.08	79.03	75.36	79.52
Protein (%)	17.71	20.96	19.57	22.17
Pectin (%)	53.20	58.80	52.10	57.53
tannin (%)	30.21	32.88	29.46	34.21
total solid (%)	26.01	29.88	27.23	31.23

Comparing the membranes, 0.05 μm membrane had advantages in the removal of macromolecular materials (5% ~ 10% higher), which is very easy to understand since selectivity in microfiltration is predominantly based on size exclusion. For 0.05 μm membrane, the synergistic relationship between decreased flux and increased retention of macromolecules and total solids suggested that there was a pore blocking effect, which hampered flux and ferulic acid transmission. Overall, 0.2 μm ceramic membrane has a higher application value given its better relationship between MF processing and permeate quality.

**Figure 2:**
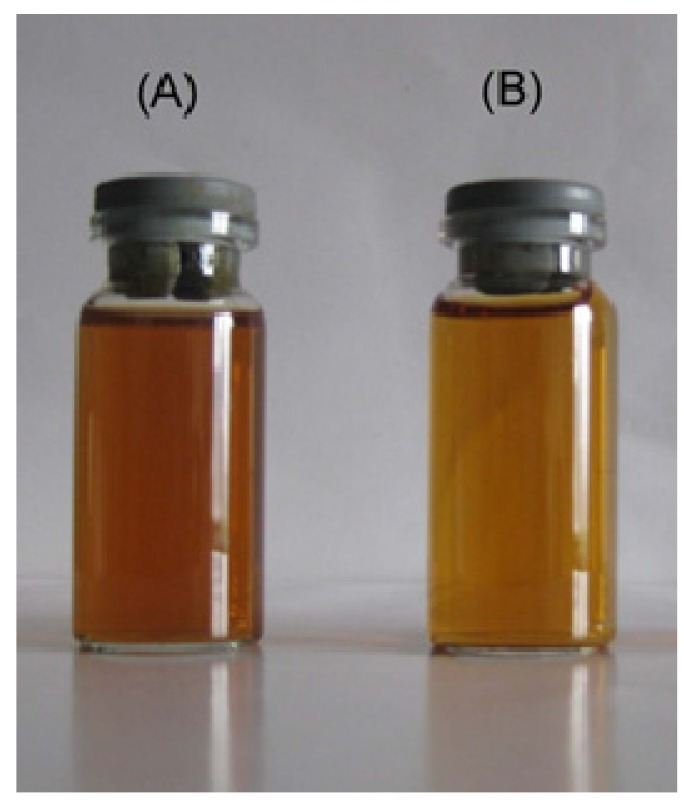
Appearance of (**A**) raw; (**B**) filtered by 0.2 μm ZrO_2_ membrane.

The refining effect of filtration by ceramic membranes could be qualitatively assessed by a simple visual test of the change in color of the raw and filtered TBL solutions. As shown in Figure 2, the colors of filtered liquor changed from brown to orange after filtration. Color is a physical-chemical parameter affected by organic matter (fibers, pectins, phenols, tannins, proteins, vitamins, *etc*.) and inorganic matter (iron, copper ions, *etc*.), as well as organometallic complexes (present in yeast or in fruit pulp) [18], and apparently, after filtration, the solution’s clarity was significantly improved.

### 2.3. Physico-chemical Analysis

The physical and chemical parameters were measured at 303 K and the experimental data are shown in Table 4. The results show that pH values changed slightly after MF. Because of the moderate macromolecular material removal, all the membranes give significant reductions in turbidity. Compared with the raw, the turbidity decreased by 32 ~ 133 times. However, TBL filtered by the 0.2 μm ZrO_2_ membrane showed higher turbidity. Permeate viscosity and density for all membranes also decreased slightly. Being different from another, the conductivity for the permeate filtrated by four membranes were increased. As an important indicator of colloidal solution, conductivity values are closely related to the charged colloidal particles such as proteins and tannins and some conductive small molecules in herbal extracts. It is assumed that during the filtration, the size of colloidal particles is decreased, so the conductivity is increased. In addition, it is suggest that high retention of colloidal particles in microfiltration would aggravate fouling for the membrane and thus would indirectly reduce the instantaneous transmittance of ferulic acid.

**Table 4:** The physical and chemical parameters of samples filtered by four membranes.

Sample	pH	Turbidity	Viscosity	Conductivity	Density(g·cm^−3^)
		(NTU)	(mPa·s^−1^)	(s·cm^−1^)	
raw	4.637	73.20	1.41	1354	0.978
0.2 μm ZrO_2_ permeate	4.641	2.29	1.36	1412	0.965
0.05 μm ZrO_2_ permeate	4.639	0.56	1.29	1575	0.962
0.2 μm Al_2_O_3_ permeate	4.651	2.13	1.35	1403	0.964
0.05 μm Al_2_O_3_ permeate	4.647	0.55	1.30	1597	0.962

### 2.4. Distribution of Fouling Resistance

**Figure 3:**
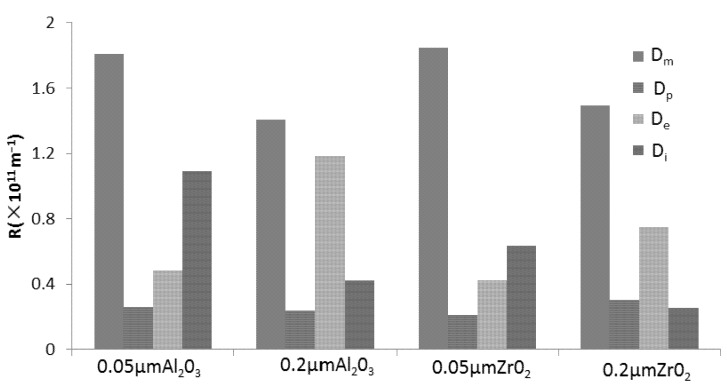
Resistance distribution of TBL filtered by ZrO_2_ and Al_2_O_3_ ceramic membrane.

The resistance of membrane process is divided into four parts: Adsorption resistance (D_e_), membrane resistance (D_m_), polarization resistance (D_p_) and internal resistance (D_i_) [19,20]. The resistance distributions of TBL filtered by the four types of membranes are shown in Figure 3. 

The data indicate that because of the lesser flux decline, membrane resistance played a dominant role in the total resistance of the all the membranes (43.2% ~ 59.1%). Comparatively speaking, for the same material, the internal resistance of the 0.05 μm membrane is larger than 0.2 μm, which indicates that for 0.05 μm membrane, the membrane pore blocking is more serious. The polarization resistances of all the membranes are both small with the values 6.8% ~ 10.8%, respectively. Therefore, despite the membrane resistance, resistance distribution is located mainly on the membrane surface for 0.2 μm membrane, while the fouling resistance is concentrated in the internal membrane for 0.05 μm membrane.

**Figure 4:**
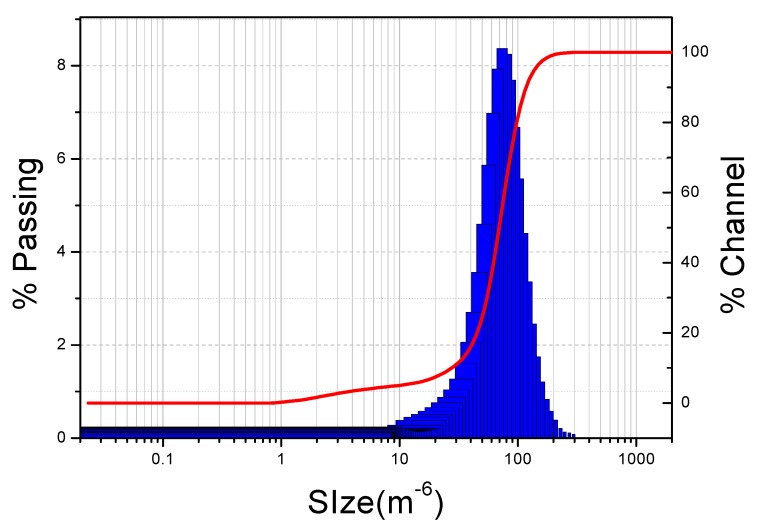
Particle size distribution of the raw TBL.

As shown in Figure 4, typical particle size of raw TBL was 73.99 μm. This could not explain the formation of internal resistance, especially for 0.05 μm whose internal resistance was dominant. It has been suggested that internal fouling is caused by deposition or adsorption of small particles and macromolecules within the internal structure of the pores [21]. In addition, some spherical proteins, branched-chain and straight-chain linear polymers in solution are very sensitive to membrane pore size and have a direct impact on the state of membrane fouling [22]. These proteins and polymers can be cut into smaller molecules by cross-flow shear stress or other mechanical force during the MF process. In the case of 0.05 μm membrane, adsorption from the interaction between solute and membrane led to changes in membrane characteristics during the early stages of MF, and the adsorbed materials can promote plugging of pores, then eventually the internal resistance would exceed adsorption resistance. In the case of 0.2 μm membrane, the pores were not blocked further because less materials are adsorbed in the early stages of MF, however the adsorption resistance would increase inevitably due to constant interaction between solute and membrane. Ultimately, the adsorption resistance becomes the chief resistance for 0.2 μm.

### 2.5. Ultrasonic Fields on Microfiltration

**Figure 5:**
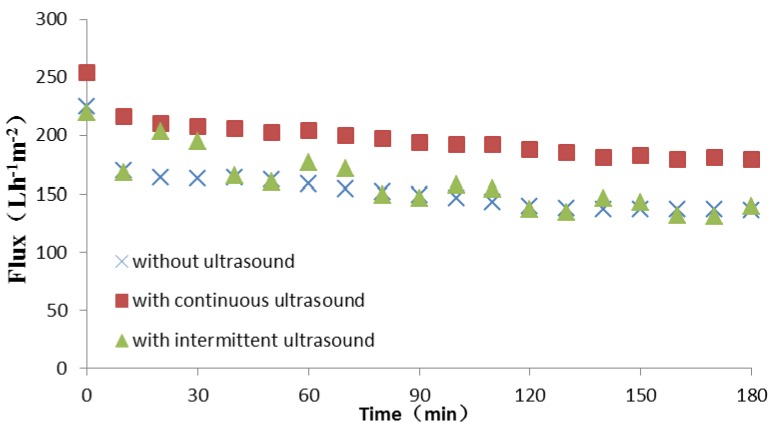
Influence of ultrasound on Tongbi liquor permeate flux.

Ultrasound, as a means of strengthening membrane process, can generate ultrasonic cavitation effect which cannot only promote macro-movement between liquid and particle but also overcome the forces between materials and membranes. As the results above, 0.2 μm ZrO_2_ was selected to be the test membrane. The typical results are shown in Figure 5. 

As shown, the permeate flux increase significantly while adding a continuous ultrasound during microfiltration. The steady permeate flux reached 179 L·h^−1^·m^−2^, an increase by 31.9% compared to the flux without ultrasound. However, the permeate flux fluctuated with cycles of the ultrasonic periodic switch. In this experiment, the approach of intermittent ultrasound was carried out that microfiltration start without ultrasound for 20 min and then opened or closed the ultrasonic instrument every 20 min. As can be seen from the curve of flux with intermittent ultrasound, the permeate flux gently declined at the first 20 min. However, it sharply increased to 203 L·h^−1^·m^−2^ at the moment of opening ultrasonic instrument, and then decayed. On the contrary, the moment ultrasonic instrument was shut down the permeate flux rapidly fell to 165 L·h^−1^·m^−2^. Subsequently, the improvement of flux was not significant as the first time when the ultrasonic instrument was opened for the second time. Ultimately, its steady flux reached 132 L·h^−1^·m^−2^ which is close to the flux without ultrasound.

This different effect on membrane process is related to the dual role of the strong shock waves and micro-jets produced by ultrasonic cavitation. On the one hand, it can strengthen the eddy diffusion and shock, erode, and peel off liquid-solid interface so as to enhance the process of membrane microfiltration [23,24]. On the other hand, the materials within membrane pores are diffused to a large extent because of a minor disturbance caused by ultrasonic cavitation effects. For this experiment, ultrasound produced a number of small particles which were not easy to plug ceramic membrane pores during continuous ultrasound. However, during intermittent ultrasound, these small particles were prone to being pressed into membrane pores when ultrasonic instrument was shut down, resulting in internal fouling. Consequently, the intermittent ultrasonic effect on permeate flux is less obvious and continuous ultrasound is more applicable. 

**Figure 6:**
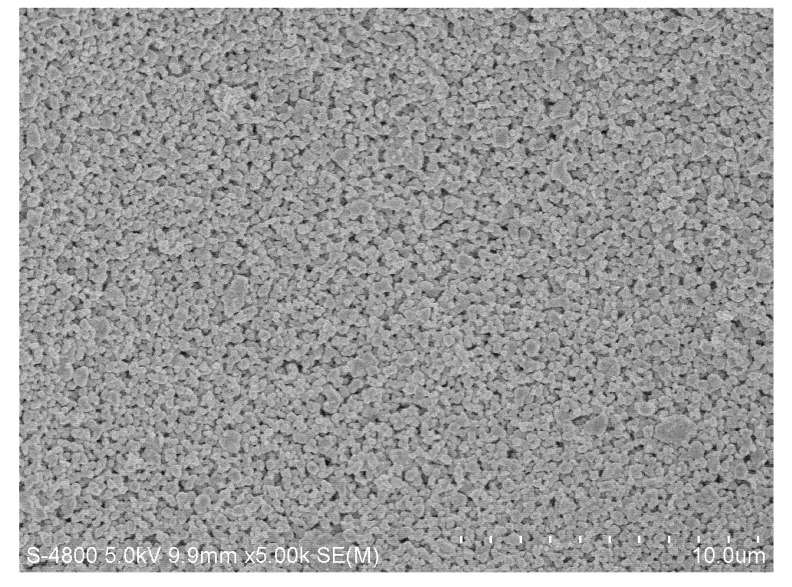
Photograph of the ZrO_2_ membrane, magnification 5,000×.

**Figure 7:**
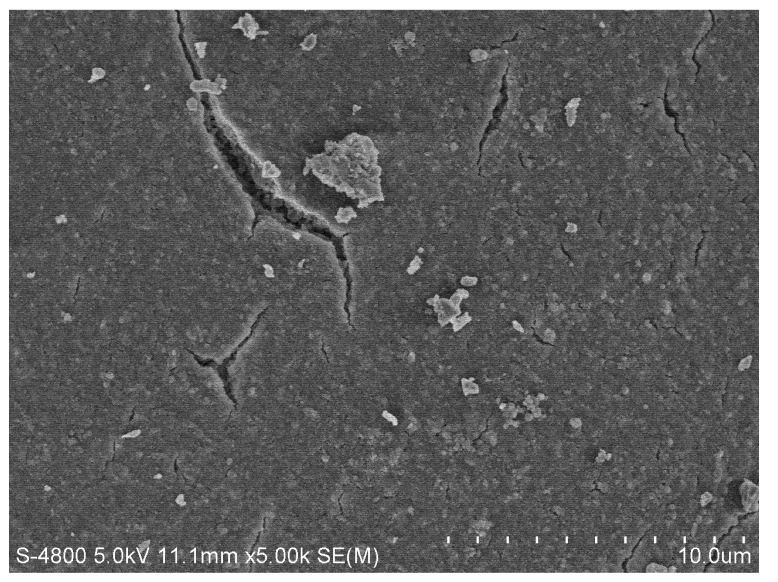
Photograph of the ZrO_2_ membrane with continuous ultrasound after filtration of the TBL(with continuous ultrasound), magnification 5,000×.

**Figure 8:**
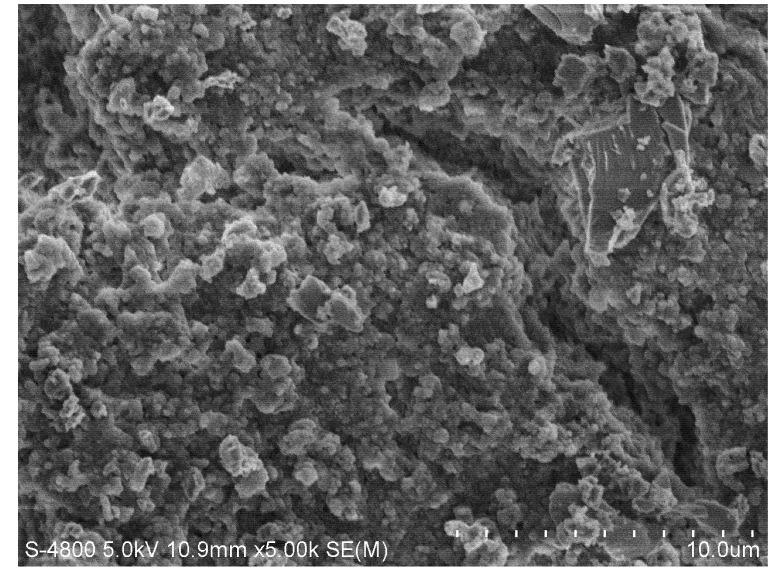
Photograph of the ZrO_2_ membrane without ultrasound after filtration of the TBL, magnification 5,000×.

**Figure 9:**
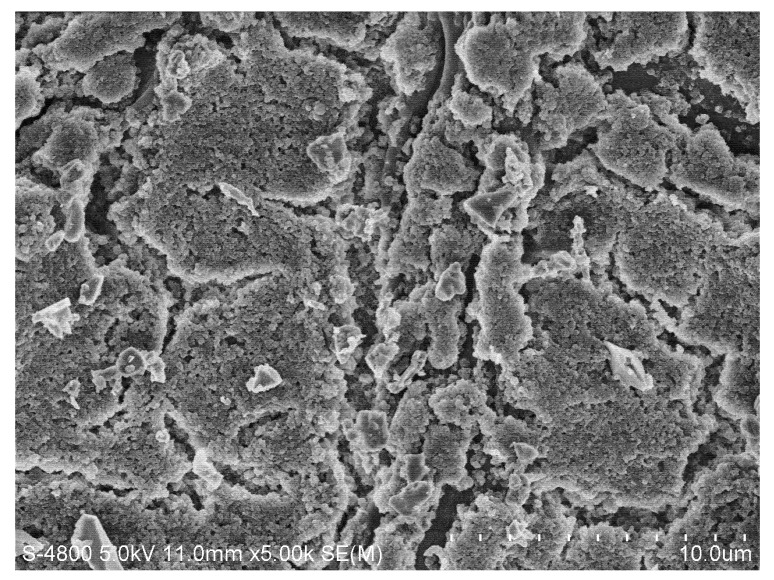
Photograph of the ZrO_2_ membrane with intermittent ultrasound after filtration of the TBL (with intermittent ultrasound), magnification 5,000×.

Photographs of the membrane surfaces were obtained with the use of a scanning electron microscope (SEM) in 5,000× magnification. The SEM micrographs show that, comparing to the membrane without pollution (Figure 6), the cake layer on the membrane surface was much thinner with continuous ultrasound (Figure 7) than without ultrasound (Figure 8) and with intermittent ultrasound (Figure 9) after filtration. The observations of the membranes surface revealed that they are covered with the residual cake, but the membrane with continuous ultrasound after filtration is obviously less polluted. 

## 3. Experimental

### 3.1. Preparation of Tongbi Liquor

The TBL was prepared by pouring *Radix aconiti kusnezoffii praeparata* (162.5 g), *Angelica sinensis* (53.95 g), *Syzygium aromaticum* (53.95 g), *Rhizoma alpiniae officinarum* (53.95 g) and *Cortex Acan thopanacis Gracilistyli* (53.95 g) into 6,500 mL of Kaoliang spirit and boiling at 40 °C for 14 days After filtering with gauze to remove residue, the liquor was then centrifuged at 3,000 rpm for 30 min, and the supernatant was obtained.

### 3.2. Cross-flow Microfiltration

The filtration was performed in a cross-flow microfiltration lab-scale plant with tubular ceramic membrane (Nanjing Jiusi High-Tech Co. Ltd., Nanjing, China) of 0.2 μm or 0.05 μm pore size with the dimension of 12 mm external diameter, 8.0 mm inner diameter and 220 mm long. The membrane is asymmetric, with the support with ceramic material which the average pore size is 5 μm. The operating setup consists of a 7 L stainless-steel feed tank (jacketed for temperature control), a flow-meter, a centrifugal pump, a membrane module, and the accessories of pressure gauges, valves, and pipes. Filtration was processed at 303 K, Re = 19,300 (which corresponded to 3 m/s feed flows), and 0.15 MPa transmembrane pressure. In order to observe the dynamic changes in the MF process, permeate was collected when the cumulative permeate volume account for 1/9, 2/9, 3/9, 4/9, 5/9, and 6/9 of the raw liquor volume. The retentate was expected to return to the feed tank. Permeate was collected and measured every 60 s by an electronic microbalance (TE4100 Sartorius Co. Ltd., Gottingen, Germany) with a data acquisition system.

### 3.3. Tongbi Liquor Characterization

#### 3.3.1. Particle Size Distribution of Raw TBL

The determination of particle size distribution of raw TBL was performed on a MICROTRAC S3500 particle size analyzer (Microtrac Inc., Montgomeryville, PA, USA).

#### 3.3.2. Indicator of the Active Ingredients

Ferulic acid is the active ingredient of Angelica, which plays a dominant role in anti-platelet aggregation, inhibition of platelet 5-hydroxytryptamine (5-HT) release, inhibition of platelet thromboxane A2 (TXA2) generation, analgesia, *etc.* [25]. Therefore, ferulic acid is the active ingredient of Angelica and was chosen as an indicator for the active ingredients of TBL. It is then analyzed by HPLC according to the method described by Zhang [26].

#### 3.3.3. Physico-chemical Analysis

TBL was analyzed for pH, turbidity, viscosity and conductivity. The pH was measured on a PHSJ-4A lab pH meter (Shanghai Precision Scientific Instrument Co., Shanghai, China). Turbidity was determined with a SZD-2 intelligent scattered light turbidimeter (Shanghai Water Equipment Engineering Co., Shanghai, China). A Brookfield DDJ-I viscometer (Brookfield Inc., Middleboro, MA, USA) was used to measure viscosity at 303 K. Conductivity was measured using a DDSJ-308A conductivity meter (Shanghai Water Equipment Engineering Co., Shanghai, China).

#### 3.3.4. Macromolecular Materials and Total Solids Analysis

The four kinds of macromolecular material analyzed in this work were starch, protein, pectin and tannins. Starch was measured according to the method of enzyme digestion described by Ning [27]. Protein and pectin were analyzed by the Bradford method and AAS method, respectively [28]. The tannins and total solids content were determined following the procedure described by Chinese Pharmacopoeia [29].

### 3.4. Experiments of Ultrasonic Fields on Microfiltration

**Figure 10:**
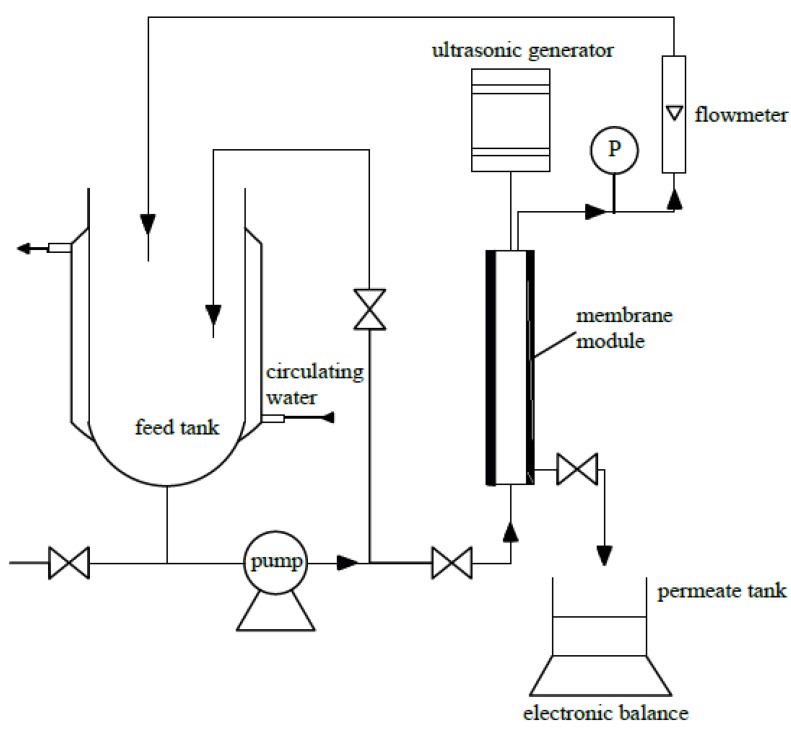
Schematic diagram of crossflow microfiltration system.

The test apparatus shown in Figure 10 was a crossflow filtration unit joining a ultrasonic generator. The volume of the feed tank was 7 L. The membrane module has dimensions of channel length of 220 mm and channel inner diameter of 8 mm. The microfiltration was run at a constant temperature of 303 K, operating pressure of 0.15 MPa and Re = 19,300 (which corresponded to 3 m/s feed flows). The effects of ultrasound on the permeate flux were investigated through microfiltration of the TBL using the 0.2 μm ZrO_2_ membrane.

### 3.5. Experiments of Scanning Electron Microscope

A Scanning Electron Microscope (SEM) (Hitachi s-4800 Co., Tokyo, Japan) was used to examine the cake layer on the membrane surface. To obtain dry membranes for surface observation by scanning electron microscope (SEM), the membranes were freeze-dried with a freeze dryer (Marin Christ Co., ALPHA1-6, Osterode, Germany). The dry membranes were fractured and treated with Au sputtering. The surfaces of the membranes were observed by SEM with an accelerating voltage of 5.0 kV.

### 3.6. The Theory of Distribution of Fouling Resistance

Darcy’s law was used as the basis to draw the following filtration flux expression. The permeate flux decline can be analyzed in terms of resistance in series. These resistances include:

- an external fouling called reversible membrane resistance which is due to the cake deposition on the membrane surface and the concentration polarization of particles;

- an irreversible resistance, R_i_ (m^−1^), due to particle and macromolecule deposition and adsorption into the membrane pores;

- and the membrane resistance.


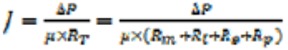
(1)

The serial resistances due to each fouling mechanism are calculated from the flux loss under different operating conditions. The following fluxes (J_0_, J_1_, J_2_, J_m_) were measured:

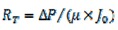
(2)
where J_0_ is the flux with TBL under a given set of operating conditions.

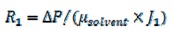
(3)
where J_1_ is the pure water flux for a membrane which has been fouled by permeation of the actual TBL effect.

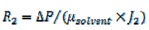
(4)
where J_2_ is the pure water flux measurement after mechanical removal of the deposited cake layer on the membrane surface, gently by a sponge.

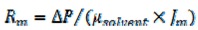
(5)
where J_m_ is the pure water flux for a new membrane.

After derivation:


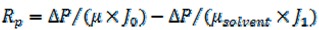
(6)


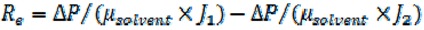
(7)


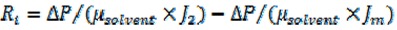
(8)

The flux loss ratios are not the serial resistances normally associated with the series resistance model in Equation (1). However, the flux loss ratios can be expressed as a function of the resistances determined with, Equations (6), (7) and (8). The Eqs can be written as:


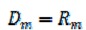
(9)


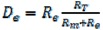
(10)


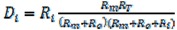
(11)


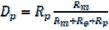
(12)

According to aforementioned states, the permeate flux was obtained in each step and using Darcy’s law, the resistances were calculated. All experiments were done in duplicate and results were reproducible with a ±5% error.

## 4. Conclusions

In this work, microfiltration of TBL was carried out using ZrO_2_ and Al_2_O_3_ ceramic membranes with pore size of either 0.2 μm or 0.05 μm. We found that 0.2 μm ZrO_2_ membrane was more appropriate because it performed better in terms of permeate flux, membrane fouling, permeation of ferulic acid, level of clarity, and removal of macromolecular materials. Using a 0.2 μm ZrO_2_ membrane, the permeate flux reached 135 L·h^−1^·m^−2^ and its fouling resistance concentrated on the membrane accounted for 53%. TBL filtered by a 0.2 μm ZrO_2_ membrane changed from turbid to transparent. Other advantages of 0.2 μm ZrO_2_ membrane over the other included a higher permeation of ferulic acid (65.53%) and better removal retention of macromolecular materials and total solids. In the experiment of ultrasonic fields on microfiltration, the permeate flux increased significantly when adding a continuous ultrasound, its steady permeate flux reached 179 L·h^−1^·m^−2^. However, the intermittent ultrasound might aggravate internal fouling. This work may be useful for refinement of Chinese herb extracts by using microfiltration processes.

We demonstrate here the use of ceramic membrane as a feasible alternative to conventional methods of filtering TBL. However, further studies on diminishing membrane fouling and enhancement of permeation of active ingredients, *etc*. are critical to the development of membrane filtration technology.
